# Global Gene Expression Profiling Of Human Pleural Mesotheliomas: Identification of Matrix Metalloproteinase 14 (MMP-14) as Potential Tumour Target

**DOI:** 10.1371/journal.pone.0007016

**Published:** 2009-09-15

**Authors:** Stefania Crispi, Raffaele A. Calogero, Mario Santini, Pasquale Mellone, Bruno Vincenzi, Gennaro Citro, Giovanni Vicidomini, Silvia Fasano, Rosaria Meccariello, Gilda Cobellis, Simona Menegozzo, Riccardo Pierantoni, Francesco Facciolo, Alfonso Baldi, Massimo Menegozzo

**Affiliations:** 1 Gene Expression Core - Human Molecular Genetics Laboratory, Institute of Genetics and Biophysics, Naples, Italy; 2 Bioinformatics and Genomics Unit, Department of Clinical and Biological Science, University of Turin, Turin, Italy; 3 Department of Thoracic Surgery, Second University of Naples, Naples, Italy; 4 Department of Biochemistry, Sect. Pathology, Second University of Naples, Naples, Italy; 5 Campus BioMedico University, Section of Oncology, Rome, Italy; 6 SAFU Department, CRS, Regina Elena Cancer Institute, Rome, Italy; 7 Department of Experimental Medicine, Second University of Naples, Naples, Italy; 8 Campania Regional Operating Center (COR) of the National Mesothelioma Registry (ReNaM) - Department of Experimental Medicine, Second University of Naples, Naples, Italy; 9 Department of Thoracic Surgery, Regina Elena Cancer Institute, Rome, Italy; Oregon Health & Science University, United States of America

## Abstract

**Background:**

The goal of our study was to molecularly dissect mesothelioma tumour pathways by mean of microarray technologies in order to identify new tumour biomarkers that could be used as early diagnostic markers and possibly as specific molecular therapeutic targets.

**Methodology:**

We performed Affymetrix HGU133A plus 2.0 microarray analysis, containing probes for about 39,000 human transcripts, comparing 9 human pleural mesotheliomas with 4 normal pleural specimens. Stringent statistical feature selection detected a set of differentially expressed genes that have been further evaluated to identify potential biomarkers to be used in early diagnostics. Selected genes were confirmed by RT-PCR. As reported by other mesothelioma profiling studies, most of genes are involved in G2/M transition. Our list contains several genes previously described as prognostic classifier. Furthermore, we found novel genes, never associated before to mesotheliom that could be involved in tumour progression. Notable is the identification of MMP-14, a member of matrix metalloproteinase family. In a cohort of 70 mesothelioma patients, we found by a multivariate Cox regression analysis, that the only parameter influencing overall survival was expression of MMP14. The calculated relative risk of death in MM patients with low MMP14 expression was significantly lower than patients with high MMp14 expression (P = 0.002).

**Conclusions:**

Based on the results provided, this molecule could be viewed as a new and effective therapeutic target to test for the cure of mesothelioma.

## Introduction

Malignant mesothelioma (MM) is a rare, highly aggressive tumour that arises from the surface serosal cells (pleural, peritoneal and pericardial cavities). Epidemiological and clinical data show that there is an association between asbestos exposure and MM development [1], even if the exact mechanism whereby asbestos induces MM is unknown [2], [3]. Western countries delayed in applying prevention measures connected to the risk of asbestos and this will produce a global increase of MM in the next years. This pathology has a long latency but a very short survival; until now the small number of drugs used for MM therapeutic treatment, does not seem to provide any clear advantage if used in different combinations or as monotherapy [4]. The prognosis is generally poor with a reported median survival of 4 to 12 months in either untreated or treated patients. Moreover, the reported response rate to the different therapeutic protocols ranged from 10% to 45% with no clear advantage in terms of poor survival (4–9 months). Currently, the trimodality approach - that employs extrapleural pneumonectomy followed by combination of chemoradiotherapy - is applied [5]. *Moreover, the combination of chemotherapy followed by surgery supplemented by postoperative radiotherapy in cases of incomplete resection, seems to be a promising treatment (Kaufman and Pass, 2008). Recent randomized studies on treatment of MM with combined chemotherapy demonstrate a survival benefit when a combination of cisplatin and antifolate drugs has been used (Fennell et al., 2008). A combined treatment, using the COX-inhibitors piroxicam with cisplatin, was recently tested in a murine orthotopic model of MM, showing an anti-tumour effects with survival increasing* [*6*], [*7*] *. Another promising pre-clinical study, based on a combined treatment with cisplatin and the proteasome inhibitor Bortezomib, showed an apoptosis increasing in MM cell lines* [*8*] *.*

Until now the molecular bases that induce MM development are still unknown; moreover, to make a precise diagnosis invasive techniques like thoracoscopy or biopsy are used, since no putative biomarkers have been clearly defined for this deadly disease, and actually the available MM biomarkers shows some limitations [9]. Indeed, the long incubation period of asbestos-related MM development, implies that the malignant transformation is related to different and multiple genetic changes. Microarray technology leads to perform transcriptomic analysis and correlates variations in the gene expression with cellular and physiological state; this is important because all processes determining variations in gene expression can give support to malignant transformation [10]. It is emerging that in MM the discovery of new diagnostic biomarkers is needed to achieve improved early diagnosis, in order to apply, in a selective way, multimodal therapy as in a pre-clinical stage or before definitive surgery. This makes the survival time increase (from 11 months to 5 years in epithelial MM) [11]. Moreover, the identification of tumour biomarkers could greatly facilitate surveillance procedures for all the cohorts of patients exposed to asbestos, a phenomenon common in different areas of the western countries, such as different in zones of Italy [12].

*In the last year microarray analyses, have contributed to shed light in understanding the molecular basis of MM development and progression. The global number of MM samples profiled is growing-up but yet is quite low - in comparison with other tumours - mainly because the low incidence of MM and the difficulty of retry large numbers of tumour samples.*

*These analyses reported deregulation of different genes involved in mitotic checkpoint that might have a key role in MM development and maintenance. Furthermore the identified genes were used to generate predictive gene-list.*

*A profiling of 21 MM tumours was used to generate a 27-gene neural network classifier to discriminate patients in short-term and long-term survivors* [*13*]. *A subsequent profiling of 31 MM samples was used to develop first a 22-gene list that was reduced to a non-overlapping 7-gene list used to derive prognostic predictors of 1-year survival.* [*14]*, [*15*]. *More recently two different large transcriptional profiling led to identify new genes allowing to discriminate tumour tissues from normal* [*16*], *or allowing to predict a 1-year survival* [*17*].

The goal of our study was the molecular dissection of MM tumour pathways by mean of microarray technology in order to identify new tumour biomarkers, which could be used as early diagnostic markers or as new specific therapeutic targets. To this end, we used Affymetrix technology to identify genes differently expressed between normal and MM patient pleural tissues. *Our analysis confirmed some previously described differences in expression patterns*. In addition, we identified and validated, for the first time, the different expression level of some genes during MM progression. In conclusion, our analysis allowed us to identify a series of novel progression-associated changes in gene expression, and to confirm, at the same time, a number of previously described results.

## Methods

### Tissue acquisition for microarray

Subjects selected for the analysis were 9 patients, consecutively treated at the Department of Thoracic Surgery of the Second University of Naples from 2004 to 2005, underwent a standard thoracotomy for therapeutic reasons (Table 1).

**Table 1:** Clinical characteristics of patients enrolled for the microarray analysis.

Patient	Age	Sex	Histological subtype
1	69	Male	Epithelioid subtype
2	59	Male	Biphasic subtype
3	61	Male	Epithelioid subtype
4	59	Male	Biphasic subtype
5	59	Male	Biphasic subtype
6	78	Male	Biphasic subtype
7	70	Female	Epithelioid subtype
8	71	Male	Epithelioid subtype
9	55	Male	Biphasic subtype

MM tissue was obtained from those patients with a confirmed pathological diagnosis and who had not received prior therapy. Intraoperative malignant mesothelial samples and nodules were dissected from associated fat and connective tissue, but no microdissection was performed. H&E staining was performed to verify the presence of neoplastic cells and to determine the histological subtype. Samples were stored in RNAlater (Ambion) following the manufacturer's protocol until RNA extraction. Control pleural tissue was obtained from patients undergoing resection for a non-neoplastic disease. Each patient gave a written informed consent in accordance with Italian law. The study project was submitted and approved by the Ethic Committee of AOU of Second University of Naples, Italy.

### Clinical Data and Tumour Specimen Acquisition for immunohistochemistry

70 patients (see Table 2) were treated at the Second University of Naples and at Regina Elena Cancer Institute, between 2001 and 2006. Clinical data were obtained by retrospective chart review. Survival period was determined from the date of initial surgery. Indeed, surgery/biopsy was the first step of diagnosis in all patients. As a consequence surgery/biopsy and diagnosis were overlapped. Follow-up was available for all patients. Four subjects who died of causes other than MM during the follow-up period were excluded from the study. 44 patients were treated with cytoreductive surgery, while all patients were treated with radiotherapy or chemotherapy. The same staging procedures were used for all the patients. Tissues from 70 MM specimens (45 epithelioid, 11 sarcomatoid and 14 mixed mesotheliomas) obtained from open biopsies or pleurectomies were collected and fixed in 10% formalin before being embedded in paraffin. The formalin-fixed, paraffin-embedded samples were sectioned at 5 µm and stained with hematoxylin and eosin. The histological diagnosis was reexamined by two pathologists (A. B. and P.M.) according to the WHO. In some cases, immunohistochemical tests were included for verification of the diagnosis as described [18]. In addition, the most representative blocks were selected to be cut into new 5 µm-thick sections for immunohistochemical studies. Each patient gave a written informed consent in accordance with Italian law. The study project was submitted and approved by the Ethic Committee of AOU of Second University of Naples, Italy and of Regina Elena Cancer Institute.

**Table 2:** Characteristics of the patients enrolled in the study.

Median age (range)	65 (45–81) years
Gender (female vs male)	29 vs 41
*Surgery*	
Yes	44 (64%)
No	26 (36%)
*T status*	
T1	4 (6%)
T2	13 (19%)
T3	23 (33%)
T4	4 (6%)
TX	26 (36%)
*N status*	
N0	27 (39%)
N1	3 (4%)
N2	14 (21%)
NX	*26 (36%)*
*Histology*	
Epithelioid	45 (64%)
Mixed	14 (21%)
Sarcomatoid	11 (15%)
*MMP-14 score*	
Low	19 (27%)
Medium	32 (46%)
High	19 (27%)

### Immunohistochemistry

Sections from each specimen were cut at 5 µm, mounted on glass and dried overnight at 37°C. All sections were then deparaffinized in xylene, rehydrated through a graded alcohol series and washed in phosphate-buffered saline (PBS). PBS was used for all subsequent washes and for antibody dilution. Endogenous peroxidase activity was blocked by 5% hydrogen peroxide. For immunohistochemistry, tissue sections were heated twice in a microwave oven for 5 min each at 700 W in citrate buffer (pH 6.0) and then processed with the standard streptavidin-biotin-immunoperoxidase method (DAKO Universal Kit, DAKO Corp., Carpinteria, CA, USA). Rabbit polyclonal anti-MMP14 (Affinity BioReagents, Golden, CO, USA) was used following the manufacturer's indications (working dilution 1∶200). Diaminobenzidine was used as the final chromogen, and hematoxylin as the nuclear counterstain. Positive controls included in each experiment consisted of tissue previously shown to express the antigen of interest. All samples were processed in the same run as one batch. Two observers (A.B. and P.M.), blinded to treatment conditions, evaluated the staining pattern of the proteins separately and quantized the protein expression in each specimen by scanning the entire section and estimating the number of positive cells at the high-power-field 10×20 and described as: low (from 1% to 20% of positive cells); medium (from 21% to 40% of positive cells); and high (more than 40% of positive cells). The level of concordance for the final scores, expressed as the percentage of agreement between the observers, was 92.5% (37 over 40 cases). In the remaining three specimens, the score was obtained after collegial revision and agreement. An univariate survival analysis for each prognostic variable on overall survival was estimated according to the Kaplan-Meier method [19]. The terminal event was death attributable to cancer. The statistical significance of the differences in survival distribution among the prognostic groups was evaluated by the log-rank test [20]. p values <0.05 was regarded as statistical significant in two tailed tests. SPSS software (version 10.00, SPSS, Chicago) was used for statistical analysis.

### GeneChip array sample preparation

Total RNA was extracted from each of the tumour and control samples using the RNeasy Mini kit (Qiagen, Valencia, CA). Biotinylated cRNA target was produced starting from 3 µg of total RNA in according to Affymetrix (Santa Clara, CA) instructions and for each sample 15 µg were fragmented to a length of 20–200 bp before hybridization to Genechip HGU133 plus2.0 arrays.

All the hybridization, washing, staining and scanner procedures were done using a Genechip Affymetrix station (Fluidics station 450, GeneChip Scanner 3000) as recommended by manufacturer. Laser scansion generated digitized image data files and CEL file that were used for the subsequent statistical analysis.

### GeneChip array data analysis

Microarray quality control and statistical validation were performed using Bioconductor [21]. The presence of hybridization/construction artifacts was evaluated with the fitPLM function (Bioconductor package affyPLM). The probe (PM) intensity distribution was evaluated using hist function (Bioconductor package affy). Probe set intensities were obtained by means of GCRMA [22] and normalization was done according to quantiles method [23]. The number of genes evaluated was reduced by applying an interquartile (IQR) filter (24953 probe sets with IQR ≥0.25 were retained) followed by an intensity filter (17716 probe sets with expression signal ≥100 in at least 25% of the arrays were retained) to remove the non significant probe sets (i.e. those not expressed and those not changing) [24]. Differential gene expression between MM and wild-type samples was detected using an empirical Bayes method [25] together with a false discovery rate (FDR) correction of the P-value [26]. Specifically, 470 probe sets (386 genes) were selected using a corrected p-value threshold of 0.05 and fold change threshold of |log_2_(fc)| ≥1. OneChannelGUI graphical interface package was used to run any of the described analysis [27]. Ingenuity Pathway Analysis (Ingenuity Systems, http://www.ingenuity.com/) was used to functionally annotate genes according to biological processes and canonical pathways and to search for potential biomarkers. Hierarchical clustering analysis of the biomarkers expression data was done using Tmev (http://www.tm4.org/mev.html).

Microarray data reported in the manuscript was described in accordance with MIAME guidelines. Microarray data were deposited on GEO database (http://www.ncbi.nlm.nih.gov/projects/geo/) as GSE12345 series.

### Quantitative Real-Time PCR analysis

Total RNA (2 µg) from normal and tumour samples was converted to cDNA using High- Capacity cDNA Reverse transcription kit (Applied Biosystem) under conditions described by the supplier. cDNA from this reaction was used directly in the qRT-PCR analysis. Gene specific primers for the selected genes (*MMP14:* Forward 5′ TCAAGGAGCGCTGGTTCTG, Reverse 5′ AGGGACGCCTCATCAAACAC; *TOP2A*: Forward 5′ TGCCAATGCTTCCAAGTTACAA, Reverse 5′TGTATGTCTGGGTCCATGTTCTG; *MDK*: Forward 5′ CAAAGGCCAAAGCCAAGAAA, Reverse 5′ GATTAAAGCTAACGAGCAGACAGAAG; *AURKA*: Forward 5′ CACCTTCGGCATCCTAATATTCTT, Reverse 5′ GGGCATTTGCCAATTCTGTT; *TGFBR3*: Forward 5′GCTGCCCAACTAAAAGGAAAAC, Reverse 5′ GAGGCTTTGCTCTGATTTCGA; *EDG1*: Forward 5′ GAGCGAGGCTGCGGTTT Reverse 5′ GGTGGTTCGATGAGTGATCCA) were designed using Primer Express 2.0 software (Applied Biosystems, Foster City, CA) and, when possible, the same coding target region identified by the Affymetrix probe was amplified; otherwise, primers were designed on the coding sequence. The specificity of each target amplicon was assessed by dissociation curve analysis and all amplicons were spanning over exon-exon regions to avoid genomic amplification. Quantitative PCRs were done on an ABI PRISM 7900HT Sequence Detection System (Applied Biosystems) in 96-well plates using a final volume of 20 µL and the following cycle conditions: 50°C for 2 minutes, 95°C for 10 minutes, and then 40 cycles of 15 seconds at 95°C and 1 minute at 60°C. All quantitative PCR mixtures contained 1 µL cDNA template (corresponding to 20 ng retrotranscribed total RNA), 1x Sybr Green PCR-Master-Mix (2x; Applied Biosystems) and 150 µmol/L of each target-specific primer. For each experiment, a no-template reaction was included as negative control. The expression of each target gene was evaluated by a relative quantification approach [28], using Glyceraldehyde 3-phosphate dehydrogenase (*GAPDH*: Forward 5′ GGAGTCAACGGATTTGGTCGTA, Reverse 5′ GAATTTGCCATGGGTGGAAT) as internal reference. Internal control was selected within not differentially expressed genes in this experiment. The Ct values of triplicate RT-PCR reactions were averaged for each gene in each cDNA sample. For each sample assayed, the level of gene expression for the corresponding gene of interest was calculated against that of the reference gene (GAPDH). Control sample were used as calibrator and each target genes was accepted as differential expressed when the ΔΔC_t_ absolute value was >1, which correspond a 2-fold change in transcript abundance. The standard deviation was calculated for samples within each tissue group.

## Results

### Clinical Characteristics of Patients enrolled for microarray analysis and Gene expression profile

Nine patients with MM underwent surgical resection and debulking for pleural MM. Their clinical characteristics are shown in Table 1. The average age at time of operation was 64.5 years. The average smoking history was 30.3 pack-years. Seven patients had some history of asbestos exposure. Six patients underwent a pleurectomy. Pathology was either epithelioid (45%), or biphasic (55%).

**Figure 1:**
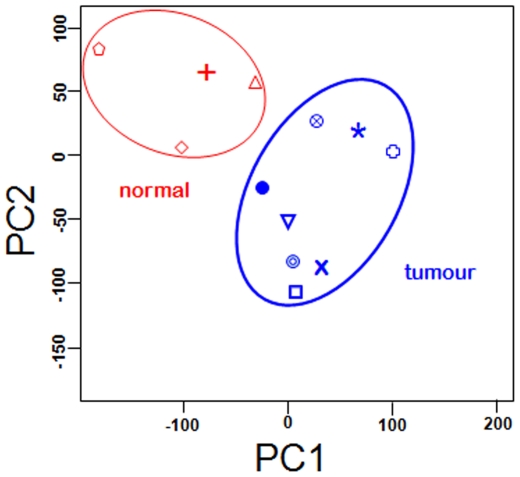
PCA of the full datasets for normal and tumour samples. PCA shows homogeneity of the experimental group, coupling the normal (red) and tumour (blue) samples in two distinct clusters.

We performed a genome wide transcription profiling to identify prognostic early molecular marker. Specifically, we compared 9 MM versus 4 normal donors pleura (see materials and methods). Transcription profiling was performed using HG U133 plus 2.0 GeneChips and data were analyzed using the oneChannelGUI Bioconductor package [27]. Principal Component Analysis (PCA) (Figure 1) clearly indicates the presence of a certain amount of differences between mesothelioma and normal pleura since they group into two distinct clusters, *separating tumour from control samples. The PCA results showed a molecular homogeneity shared by tumour samples, and prompted us to proceed in the subsequent statistical analysis without discriminating between epithelioid and biphasic subclasses. The discrepancy between transcription data and histological classification is due to the fact that MM histology shows an important phenotypic variability and the classification is based on the relative amount of epithelial and spindle cells and can therefore can be very dependent on the pathologist that performs the analysis.*

The complexity of the data set was reduced removing the non significant probe sets (i.e. those not expressed and those not changing). This filtering procedure reduced the initial set of 54675 probe sets to 17716 that were used for further analysis. A moderated t-test [25] was used to detect differential expression between tumour and normal tissues. Specifically, 386 genes (470 probe sets) were selected using a BH corrected p-value ≤0.05 and |log_2_(fc)| ≥1.

### Validation of selected gene expression

To independently test the validity of the differential expression signatures determined by microarray analysis, we measured the expression patterns of some of the putative biomarkers. We assessed gene expression of 6 representative genes in all tissue samples using quantitative real-time polymerase chain reaction (qRT-PCR). In all cases, the qRT-PCR results of the analyses confirmed the differences detected in our microarray analysis (Table 3).

**Table 3:** qRT-PCR validation of microarray results.

	fold change	
Gene	arrays	q-RT-PCR
MMP14	6.189952	5.012825
TOP2A	5.568903	3.597234
MDK	3.595405	4.189205
AURKA	3.564461	2.00000
TGFBR3	−3.74758	−3.0022
EDG1	−2.02870	−1.26722

### Analysis of specific pathways and genes

**Figure 2:**
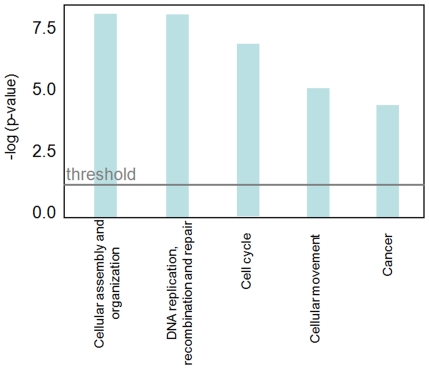
Top enriched Biofunctions as determined by Ingenuity analysis. The top five biological functions found enriched in the set of transcripts modulated in tumour mesothelioma samples detected by Ingenuity.

Functional pathway analysis of the differentially expressed genes was performed using “Ingenuity Pathways Analysis” (IPA, http://www.Ingenuity.com) a web-delivered application that enables to discover and analyze functional relation between differentially expressed genes. For each probe set, IPA generates a metadata file containing information on how to map the given dataset onto molecules associated with specific disease, cellular functions and canonical pathways; thus, it is possible to obtain indications on the cellular processes involved in the disease. IPA analysis was performed analyzing a file containing the global gene list, obtained from filtering procedures (17883 probe set), that includes statistical validated genes (470 probe sets, 386 genes). A complete list of the 386 genes differentially expressed in tumour samples is found in Table S1. This analysis highlighted that cell cycle, cellular proliferation, DNA damage and cancer are the most consistent functional classes found enriched within the set of differentially expressed genes (Figure 2). The complete gene list of the most relevant networks is reported in Table S2.

### Condensin Complex and kinesin family

Among genes related to DNA replication, in our data set we found as up-regulated components of the condensin complex (e.g. BRRN1, CNAP1, NCAPD3) and members of the kinesin family (e.g. KIF14, KIF23, KIFC1). These genes have not been previously described in MM. The two condensin complexes (I and II) play an essential role in mitotic chromosome assembly and segregation. The complexes make distinct contributions to metaphase chromosome architecture defects [29], [30] and they are essential to cell division because their knockdown generates mitotic spindle defects [31]. Thus, condensin over-expression could contribute to genome instability. The kinesin family groups microtubule-based motor proteins that play important role in multiple cellular processes including intracellular transport and cell division. Recently, elevated kinesins expression was found in cancer and associated with poor-prognosis [32]. Data *in vitro* seems to confirm their role in cancer, since their knockdown can decrease tumourigenicity [33]. Furthermore, this family is a substrate for AURKA, that we found over-expressed in our experimental setting (see later), and whose over-expression has been correlated with chromosomal instability and clinically aggressive disease [34].

### Cancer and cell-death network

Among the up-regulated genes associated to the cancer and cell death-related network (2), we found molecules with known function in cancer progression, such as the protein kinase CDC2, that has a crucial role in cell cycle control and in cell cycle progression, and whose over-expression has been reported in MM [16]. CHEK1, instead, is a checkpoint kinase involved in DNA damage response, whose depletion leads to metaphase block [35]; its role, in MM, if any, is unknown. Other genes associated to other cancers (e.g. BUB1 in bladder, MAD2L1 in breast) [36], [37] are involved in spindle checkpoint.

We found up-regulated the maternal embryonic leucine zipper kinase (MELK), a gene associated to unfavorable survival in MM [17] and known to be associated both to MM and other cancers. MELK increased expression seems to be restricted to cancer tissue [38], [39] while its silencing causes a block of tumour cells proliferation: a result that permits to hypothesize for MELK a role as molecular therapeutic target [39].

Consistent with previously published results, we found up-regulated some genes associated to poor survival and included in different prognostic classifiers, such as BTG2 (Karmanos gene classifier and MSKCC gene classifier) [13], [17], BIRC5 and KIF4A (Karmanos gene classifier), or SEPT9 (Brigham list) [15]. Furthermore, we found WT1, a gene described as favorable for survival in MSKCC gene classifier, down-regulated in our list.

### Cell cycle regulation

Among the up-regulated genes, not previously associated to MM, we found several cyclin genes (e.g. CCNA2, CCNB1, CCNB2, CCNL2). CCN gene family contributes to cell cycle regulation. Cyclin dependent protein kinases (CDKs) regulate cell cycle transitions and are essential for cellular integrity. In fact, they play pivotal role, ranging from DNA damage and spindle assembly checkpoints - before entering mitosis - to kinetochore and centrosome maturation and separation, in regulating the timing of entrance and exit of mitosis [40]. Up-regulation of these mitotic kinases was not surprising, because it is well known their involvement in tumourigenesis, considering also the central role of the phosphorylation in mitotic checkpoints, spindle function, and chromosome segregation.

CCNA2 (Cyclin A/Cdk2) plays an important role during both G1/S and G2/M eukaryotic cell cycle transitions, activating CDC2 or CDK2 kinases. CCNA2 over-expression is frequently detected in many tumours [41] and it has been associated with poor prognosis in different cancers.

CCNB1, another key component in cell cycle control, has a role in G2/M progression, acting with CDK1 to control chromosome condensation [42]; it has been implicated in tumourigenesis and in metastasis in different cancers [43].

CCNB2 is involved in chromosomal instability and its over-expression modifies spindle checkpoint and chromosome segregation [44].

These cyclins, that were not previously associated to MM, are related to each other in the regulation of centrosome separation and in the nuclear-envelope breakdown, even if they have different roles. CCNA2 is involved in mitotic entry or completion because it is the only required for a correct timing of centrosome separation and nuclear-envelope breakdown, while CCNB1 and CCNB2 have a role in mitotic progression in a CCNA2-dependent manner [45]. Thus, their concomitant over-expression could have a role in the mesothelioma tumour progression and maintenance.

### Spindle checkpoint and cell cycle progression

The network (2) also contains genes involved in spindle checkpoint function and in cell cycle progression - two processes involved in cancer development - as Aurora Kinase-A (AURKA), a well known cell cycle regulated kinase, highly expressed both in various cancer cells and during mitosis, whose role in MM is well documented [17]. AURKA expression has prognostic value because it seems directly correlated to survival and it is frequently over-expressed in many tumours [46], [47], [48].

Remarkably, we found over-expressed DLG7, a gene recently described as a potential oncogenic target of AURKA. DLG7 is an essential component of the mitotic apparatus required for spindle microtubules organization in a complex dependending on AURKA activity, that regulates DLG7 as a downstream target [49]. DLG7 was first identified as a potential oncogene, over-expressed in human hepatocellular carcinoma (HURP hepatoma up-regulated protein) [50], having an expression profile well correlated to AURKA. This suggests that they may be coordinately regulated through stabilization of DLG7 by AURKA [51]. DLG7 protein binds microtubules affecting their organization and is required for chromosome congression and alignment, functions essential for bipolar spindle formation. DLG7 over-expression in HeLa cells results in hyperstabilization of the mitotic spindle that could promote aneuploidy and genomic instability by generating subtle defects in chromosome congression [52]. Thus, over-expression of DLG7 and AURKA in cancers and in MM suggests that mis-regulation of this complex could play a role in carcinogenesis.

### FOXO3A pathway

**Figure 3:**
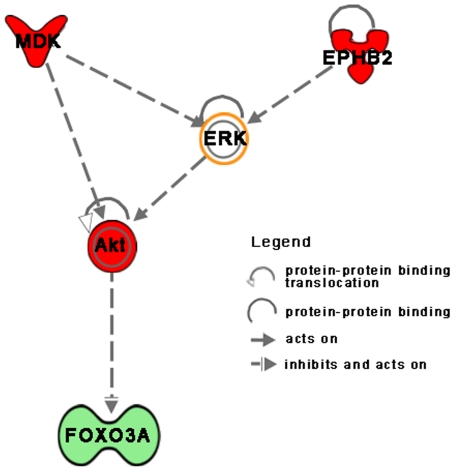
FOXO3A functional relationship. Functional pathway between genes acting on FOXO3A built by Ingenuity; the relationships are in agreement with the observed gene expressions; arrows indicate the direction and the relation type.

Among down-regulated genes it is worthy to note FOXO3A, a member of the forkhead transcription factors family that acts as a trigger for apoptosis, targeting multiple genes involved in tumour suppression [53]. It is known that RAS–ERK is an essential oncogenic signaling cascade that promotes tumour cell growth and development. A recent study demonstrated that down-regulation of FOXO3A promotes tumourigenesis and that this process involves a direct interaction of FOXO3A with oncogenic kinases, such as AKT and ERK that negatively regulate it [54]. Accordingly with this observation, we found over-expressed EPHB2 and MDK. EPHB2 is a tyrosine kinase receptor that regulates ERK and plays an important role in oncogenic processes being involved in a wide range of processes directly related with tumourigenesis and metastasis [55]. MDK is a growth factor associated with cancer development, often related to drug-resistance, that increases AKT proteins activation [56]. Looking for the functional relationship among these genes, we tried to connect them using IPA and interestingly found connections that well fit with fold change value and that finally result in FOXO3A inhibition (Figure 3).

### Mesothelioma biomarker identification

As previously described, MM is a rare but highly lethal tumour that develops after long time latency from the first asbestos exposure. Unfortunately, MM is diagnosed when the tumour has occurred and there are no more effective treatments to use. The epidemic of MM is increasing in countries that have made vast use of asbestos in the past. The epidemic curve follows that of asbestos consumption over time and the plateau is expected to be reached in the period 2010–2020 [12]. For this reason, the identification of early tumour biomarkers suitable for an early diagnosis and a proper prognosis becomes a priority for MM treatment.

**Figure 4:**
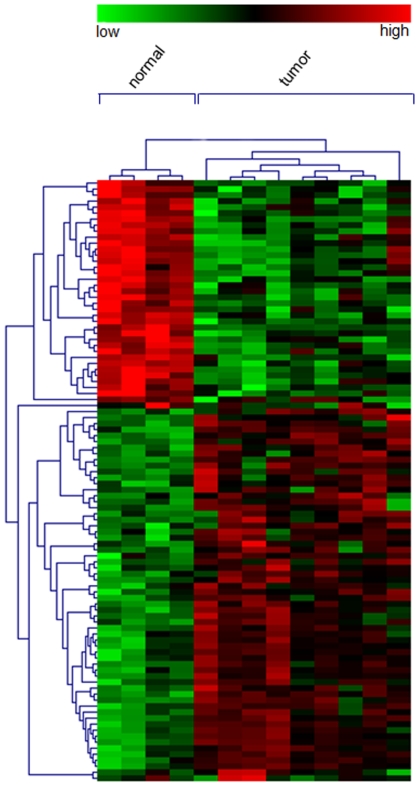
Clustering of biomarker gene list. Hierachical agglomerative clustering of the 100 transcripts found differentially expressed in tumour samples and associated to biomarkers by Ingenuity.

In order to identify genes to use as potential early diagnostic and/or prognostic biomarkers, we used our dataset as starting template to perform an IPA-biomarker analysis. IPA is a tool capable of retrieve candidate biomarkers implicated in disease processes: it determines if they could be detected in body fluids. From this analysis we obtained a gene list containing 100 putative biomarkers (1). To assess a relationship between differential expression in tumour samples and putative mesothelioma biomarker genes, we performed a hierarchical aglomerative clustering of the expression of the 100 putative biomarkers, using the TMEV tool [57] (Figure 4).

Among the down-regulated transcripts in the tumour samples, 34 were found eligible for Ingenuity analysis. The analysis highlighted the presence of a set of transcripts associated to cellular movement, molecular transport and immune response. Among the transcripts up-regulated in the tumour samples, 54 were found eligible for Ingenuity function and pathway analysis. These transcripts were associated mainly to cancer and cell cycle.

**Figure 5:**
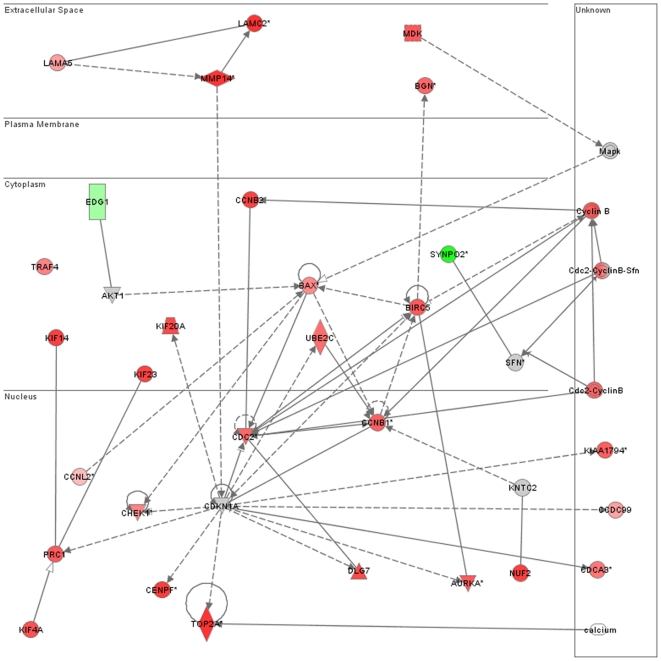
Gene network of deregulated genes associated to cell cycle and selected as putative biomarkers as determined by Ingenutity. Genes are positioned in subcellular layout. Genes in red showed increased expression in tumour samples while genes in green decreased their expression in tumour. Up-regulation of some cyclin genes is in agreement with the tumour higher proliferation rate. Relationships are marked by arrows: dashed line arrows mark indirect interactions, filled line arrows mark direct interactions.

We analyzed genes in more detail, looking for potential biomarkers that could be specific for MM. To this end, we restricted our analysis first to molecules linked both to cancer and respiratory disease and then to proteins potentially detectable in specific body fluids such as blood or plasma. Moreover, to investigate biological pathways affected by the predictive biomarker genes and to better select genes potentially usable as diagnostic marker, we built a functional pathway to search direct interactions. The retrieved interaction pathway (Figure 5) affects genes, involved in cell cycle regulation, that have increased expression in tumour samples.

As is shown in Figure 5, the detected interactions regard only few, but interesting, genes with decreased expression in tumour. Among them we found EDG1 and SYNPO2. EDG1 is a G protein-coupled receptor involved in cell-cell adhesion, reported as a novel antiproliferative protein, whose inhibition increases breast cancer cell growth [58]; SYNPO2, has been recently characterized as a tumour suppressor gene that inhibits cancer growth and metastasis both in vitro and in animal models [59].

Matching together the biomarker molecules and genes included in the pathway, we observed some interesting genes that could be further investigated as putative biomarkers. In particular, since secretory or membrane proteins might be especially useful for diagnostic aims and, possibly for therapeutic purposes, we focused our attention on MMP14 (MT1-MMP), LAMA5 and LAMC2.

MMP14 is a member of the matrix metalloproteinases (MMP) protein family, involved in the breakdown of extra-cellular matrix that has been associated with many different tumours. MMPs have a fundamental role in basement-membrane penetration during metastasis as well as in highly aggressive tumours or in late stages [60]. They can promote tumour progression through different signalling functions including apoptosis, angiogenesis and immunity. Their broad functions candidate them as drug targets [61].

*The MMP family in mammals includes different protein and peptide hydrolase that share a common domain structure. Among the member so far identified “MMP” include the membrane-type proteins that are produced as zymogen (pro-MMP), while “MT1– MMPs” include members with a membrane anchor, a feature for which they are thought to play a role in pericellular proteolysis. Overexpression of specific MMPs, as the gelatinases A (MMP-2) and B (MMP-9) and stromelysin-3 (MMP-11), have been widely associated to tumour progression and metastasis in different tumours. Furthermore MMP-2 and -9 expression has been described in MM cell lines* [*62*] *.*

MMP activity is regulated by TIMPs (tissue inhibitors of metalloproteinases), which are their endogenous inhibitors. The balance between MMPs/TIMPs regulates the extracellular matrix (ECM) turnover and remodeling during normal development and pathogenesis.

*The MMPs zymogen activation is strictly controlled by a membrane-associated event. The process strictly connects MT-MMP and MMP functions, and it has been shown that MMP14 is essential for proMMP-2 activation: pro-MMP-2 activation by MMP14/TIMP complex is realized in an environment of low TIMP concentration; furthermore TIMP 4 over-expression can reduce tumour invasiveness* [*63*] *.*

Taking in account that TIMPs role in tumour invasion and metastasis is achieved through MMPs inhibition, these proteins could be a new group of therapeutics for MMPs specific inhibition and could represent a new approach in cancer therapy [64]. According with previous published data, reporting an imbalance between matrix metalloproteinases and their inhibitors [65], [66], in our data set we found an opposite expression of a MMP (MMP14 up-regulated) and of a TIPM (TIPM4 down-regulated).

MMP14 reveals to be a very intriguing molecule that seems to be important in tumour progression by promoting cell invasion and matrix degradation. Furthermore, MMP14 has been identified as a key player during angiogenic response, a process regulated by VEGF [67] that we also found up-regulated. MMP14 shows a relationship with LAMA5 and LAMC2 (Figure 5) and this is in agreement with our analyzed phenotype. It is reported that MMP14 cleaves the LAMC2 gamma -2 chain, producing a fragment release usually increased in cancer cells. This process could be the way in which MMP14 promotes cell migration and invasion [68]. Furthermore, this proteolytic cleavage is known to convert laminin properties from cell adhesion to motility type, being a distinctive trait of invasive cancer cells.

*We investigate the presence of MMP14 up-regulation in tumours using Oncomine (* http://www.oncomine.org/ *) and GEO (* http://www.ncbi.nlm.nih.gov/geo/ *).*

*In Oncomine database there are several cancer studies in which MMP14 was found up-regulated such as ovarian, breast and colon but no MM data sets have been recorded.*

*GEO database records about three hundred gene expression cancer profiling studies in which MMP14 results to be up-regulated and frequently related to cancer progression. Looking for over-expression of this gene in MM, two studies are available, one performed in mesothelial cell lines (GDS2604 series),* [*69*] *and the other performed on MM tumor samples (GDS1220 series),* [*16*] *. On the basis of GEO data, MMP14 was found up-regulated in the tumour samples, but it was not discussed in the papers associated to the datasets.*

**Figure 6:**
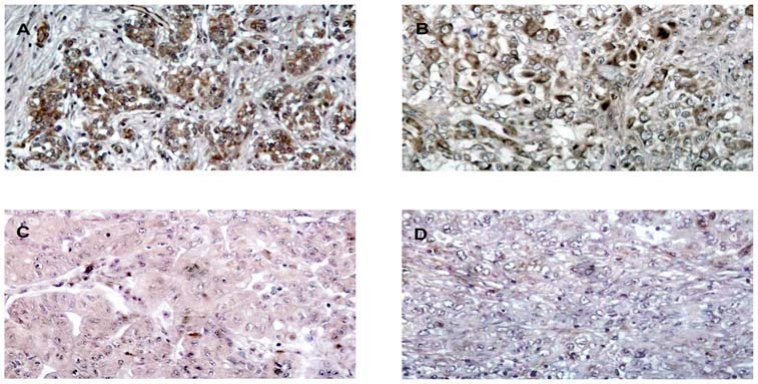
Immunohistochemical staining of MMP14 in human pleural mesotheliomas. A: Strong cytoplasmic expression (original magnification×200); B: Low cytoplasmic expression (original magnification×200); C: Negative control (original magnification×200); D: Low to undectable expression (original magnification×200).

**Figure 7:**
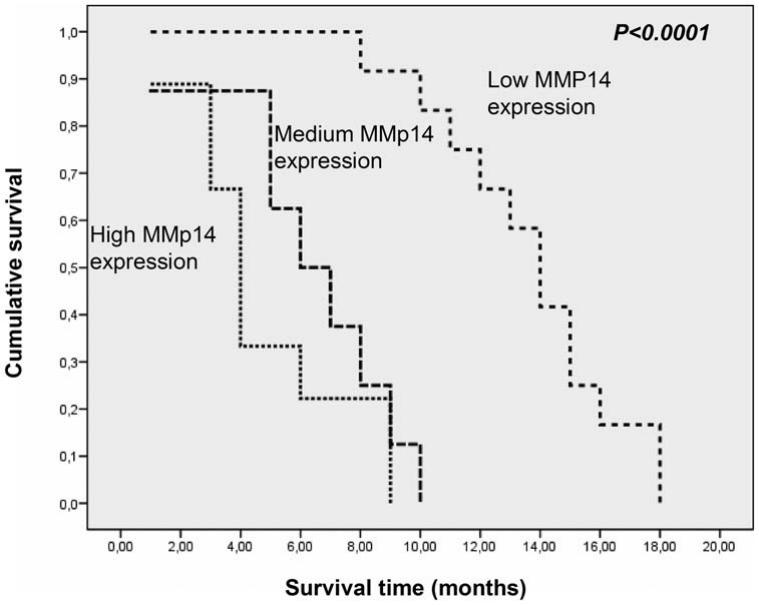
Kaplan-Meier survival plot for MMP14. The MM patients with high MMP14 expression have a significant shorter survival time respect to the low MMP14 expressors.

In order to better define the possible prognostic value of MMP14 expression in MM, we decided to investigate MMP14 expression by immunohistochemistry in a group of well-characterized MM specimens. Table 2 shows the characteristics of the patients enrolled in this study and a summary of the results from immunohistochemical analysis. MMP14 was always expressed in MM, but with different expression levels; staining was always cytoplasmatic. Figure 6 shows some typical immunohistochemical staining for MMP14. By univariate analysis overall survival was influenced by T and N status, and by histology, being the sarcomatoid pattern mostly related to a worse prognosis. Interestingly, survival was also influenced by MMP14 expression (p<0.0001). The median survival in patients with low MMP14 expression was clearly longer than in those patients with high MMP14 expression (24 months vs 5 months). On the other hand, cytoreductive surgery did not influence the overall survival in our patients' population (Table 4). In Figure 7 is depicted a Kaplan-Meier survival plot for all patients showing a statistically significant association between high MMP14 expression and poor outcome. Chemotherapy and radiotherapy did not show any impact on overall survival in univariate analysis (data not shown). Indeed, by a multivariate Cox regression analysis, the only parameter that resulted to influence overall survival was MMP14. The calculated relative risk of death in MM patients with low MMP14 expression was significantly lower than patients with high MMp14 expression (P = 0.002). All other parameters significantly associated with prognosis in univariate analysis did not influence the overall survival when evaluated by multivariate analysis, except for the T status (P = 0.01), while the histological pattern reached only a borderline significance (p = 0.079) (Table 5).

**Table 4:** Correlation with survival in univariate analysis of the anatomo-clinical and immunohistochemical parameters selected for the mesothelioma patients.

	Median Survival Time (months)	95% C.I. (months)	P value
*MMP14 score*			<0.0001
Low	26	21.15–26.84	
Medium	14	9.84–18.15	
High	5	3.68–6.32	
*T stage*			0.001
1	Not reached	-	
2	21.0	12.40–29.56	
3	11.6	2.49–17.50	
4	10.0	3.41–15.60	
*N stage*			0.020
0	17.0	7.59–26.41	
1	23.0	9.20–27.34	
2	10.0	6.88–12.87	
*Surgery*			0.398
No	14.0	10.76–17.24	
Yes	16.0	11.73–20.27	
*Histology*			0.005
Epithelioid	16.0	13.05–18.95	
Mixed	11.0	9.18–12.81	
Sarcomatoid	6.0	3.57–8.42	

**Table 5:** Prognosis of mesothelioma expressed as hazard ratios (HR) and 95% confidence intervals (CI) associated with selected immunohistochemical and anatomo-clinical parameters.

	HR value	95% C.I.	P value
*MMP14*			0.002
Low	1	-	
Medium	1.56	1.03–2.34	
High	3.56	2.48–5.90	
*T stage*			0.011
1	1	-	
2	1.23	0.56–1.44	
3	2.55	1.11–2.98	
4	2.76	2.53–3.52	
*N stage*			0.212
0	1	-	
1	0.79	0.59–1.63	
2	1.95	1.07–2.38	
*Histology*			0.079
Epithelioid	1	-	
Mixed	0.67	0.48–1.07	
Sarcomatoid	0.49	0.32–0.87	

Cox regression analysis was performed (adjusted for all the other variables) for the following factors: MMP14, T stage, N stage, Histology

## Discussion

MM is a highly aggressive neoplasm correlated to asbestos exposure and current therapies are mainly based on clinical stage and tumour histology. Even if favorable prognostic factors mostly derive from the mixed and epithelioid histology, precise identification of predictive factors or prognostic markers actually needs to be based on gene expression profiling. In fact, the trimodal chemotherapy prolongs the survival of few months and the cytoreductive therapy followed by chemotherapy/radiation combined treatment has been shown to improve survival only in early MM patients [5]. For these reasons, the identification of molecular early diagnostic or prognostic marker becomes fundamental in order to apply therapeutic protocols in the right time. Concerning these aspects, gene expression profiling of MM is a challenge promise, not only to identify prognostic indicators of clinical outcome, but as a means of characterizing specific molecular abnormalities that may underlie all the time-related poor signs of MM. *The gene expression analyses have been successfully used to classify tumours in groups that correlate either with tumour differentiation degree or with patient survival with more accuracy* [*70*] *.*

*Molecular signature of MM represents the only way to identify predictive factors or prognostic molecular markers in order to identify in advance (in pre-surgery phase) suitable long-term survivors. Furthermore the outcome prediction in MM, based only on histological classification, has been revealed to be error prone and ineffective for patients* [*71*] *. MMs show a phenotypic variability and are classified based on to the relative amount of epithelial and spindle cells. Indeed the histological heterogeneity of MM samples might correspond to the same expression profile, thus gene expression analysis could extend and refine the standard pathologic analysis.*

Our exhaustive analysis of gene expression in MM, compared to normal pleural samples, confirm the complexity of biologic events responsible of neoplastic transformation. Indeed, our data depict a complex scenario, where genes involved in different important functions for the cells (such as cellular assembly and organization, cell cycle regulation, apoptosis, cellular movement and DNA repair) are simultaneously involved to produce tumour phenotype. Therefore, this study confirms the aptitude of microarray technology in defining molecular pathways involved in MM pathogenesis and progression. In addition, our results corroborated previously observed expression patterns of a series of genes [13], [15], [17], and revealed new genes differentially expressed during MM progression.

*Our data - according to recent studies performed in MM using microarray technologies - reported deregulation of different genes involved in mitotic checkpoint that might have a key role in MM development and maintenance. Our list contains several genes previously described as prognostic classifier. Furthermore, we described novel genes, never associated before to MM, possibly involved in tumour progression.*

*Among genes related to DNA replication, we identified as up-regulated components of the condensin complex and members of the kinesin family (e.g. BRRN1, CNAP1, NCAPD3, KIF14, KIF23, KIFC1). The condensin complex is essential to normal cell division* [*30*] *and its over-expression probably contributes to genome instability. The kinesin family groups microtubule-based motor proteins that play important role in multiple cellular processes, including intracellular transport and cell division. Data in vitro seems to confirm their role in cancer* [*33*] *.*

*Kinesins and condensins are substrate for AURKA, a gene whose over-expression in MM has been correlated with an aggressive course* [*17*] *. Deregulation of AURKA is known to have a role in cancer because AURKA regulates different cell cycle events, supporting centrosomes maturation and spindle assembly and stability; its over-expression was associated in MM with chromosomal instability and clinically aggressive disease* [*17*] *. Furthermore, we found over-expressed DLG7 another essential component of the mitotic apparatus, recently described as a potential oncogenic target of AURKA. It is worthy to note that the anti-tumour effects obtained from the first generation of Aurora kinase inhibitors* [*72*] *might be tested also in MM treatment.*

*We found up-regulated several cyclin genes, that play role both in G1/S and in G2/M eukaryotic cell cycle transitions, as CCNA2, CCNB1, CCNB2, CCNL2. Cyclin A/Cdk2 (CCNA2) and CCNB1 are present in many tumours* [*41*] *and some were previously associated with poor prognosis and in metastasis* [*43]*, [73**] *. Together these cyclins have different roles in centrosome separation regulation or in nuclear-envelope breakdown, but their concomitant over-expression might have a role in MM progression and maintenance. Other molecules up-regulated with a well-known function in cancer progression, are CDC2, a protein kinase that has a crucial role in cell cycle control and in cell cycle progression* [*74*], [*75*] *and CHEK1, a checkpoint kinase involved in DNA damage response, whose depletion leads to metaphase block* [*35*] *.*

*Our data confirm the association between MELK and MM. Up-regulation of this gene has been related to unfavorable survival in MM* [*17*] *.*

*Finally, in agreement with previously published results, we found up-regulated genes associated to poor survival and included in different prognostic classifiers, such as BTG2 (Karmanos gene classifier and MSKCC gene classifier)* [*13]*, [*17*] *, BIRC5 and KIF4A (Karmanos gene classifier), SEPT9 (Brigham list)* [*15*] *, and down-regulated WT1, a gene described as favorable for survival in MSKCC gene classifier.*

The transcriptome analyses reported above, identified as up-regulated in MM almost all genes related to cellular processes whose deregulation might play a crucial role in cancer development and progression.

Logically, considering the limited number of patients analyzed, these data must be considered not conclusive; nevertheless, the information acquired could indicate some of the molecular pathways involved in MM pathology. Indeed, the role of these newly identified genes is being evaluated in further studies, aimed to analyze the protein expression pattern and the protein function *in vitro* and *in vivo* in MM cells.

In the second part of our manuscript, we focused our attention on the definition of putative biomarkers for early diagnosis and prognosis in MM. Among genes involved in mesothelial cells tumour transformation we directed our attention to molecules present in the extra-cellular space and detectable in biofluids such as blood. These new molecules could be potentially used as early diagnostic MM markers to screen individuals previously exposed to asbestos. To this aim we focused our attention on MMP14. To the best of our knowledge, this is the first paper investigating MMP14 in MM. We found MMP14 to be widely expressed in all the samples analyzed, even if with different expression levels. Interestingly, high immunohistochemical expression of MMP14 was significantly correlated with poor survival. These observations strongly suggest that MMP14 plays a role in tumour progression in MM and could be a target for MM biological therapy. In particular, MMP14 could represent a putative target for a therapy based on specific MMP14 inhibitors. To consider MMP14 as drug target, detailed analysis of its inhibition effects in patients are needed. Treatment with MMP14 inhibitors is currently under investigation and preliminary experiments have been performed on *Mmp14*-null mice [76], that showed multiple severe side effects probably related to secondary development defects and not to the protein deficiency. Thus, in order to consider MMP14 as anti-target, a more detailed MMP14 inhibition analysis in adults mice and in patients is needed. To this aim, high throughput proteomic techniques were recently applied to analyze in detail the effects on cells of a MMP14 inhibitor drug, in order to predict and possibly avoid side-effects of drug treatment in patients [77]. Further studies are urgently required both at molecular and clinical level to confirm these observations and to eventually propose MMP14 as a concrete target for therapy. Finally, in order to determine if the amount of cytoplasmic MMP14 reflects the amount that is secreted, it would be necessary to carry out prospective studies, analyzing the amount of MMP14 in the extra-cellular space of MM patients. These studies are necessary in order to eventually consider MMP-14 a biomarker for MM.

### Conclusion

MM is a rare, highly aggressive tumour related to asbestos exposure that develops after long time latency, with a very short survival after diagnosis. Since cancer therapies are more effective if used in the initial stages we analyzed MM to identify early biomarker that could be used to diagnose it in advance. Using microarray technology, we analyzed MM and normal pleural samples identifying new genes involved in tumour progression, focusing our attention on the identification of putative biomarkers for early diagnosis and prognosis in MM. To this aim we analyzed the differentially expressed genes, looking for potential biomarkers specific for MM by identification of molecules both linked to cancer and respiratory disease, and also potentially detectable in specific body fluids such as blood or plasma.

We directed our attention to molecules present in the extra-cellular space, because these molecules could be used as early diagnostic MM markers to screen individuals previously exposed to asbestos. In particular, we found MMP-14 a member of the metalloproteinase family that mediates homeostasis of the extra-cellular environment. The protein mediates the breakdown of extra-cellular matrix a process needed for basement-membrane penetration during metastasis and its over-expression has been associated with many different tumours.

We found that expression of MMP-14 has a prognostic value in a group of MM patients showing that high immunohistochemical expression of MMP14 was significantly correlated with poor survival.

*A prospective trial is undergoing in order to confirm the prognostic value of MMP-14 expression in MM patients. Finally, to better determine the role of MMP-14 as MM biomarker, our research group is actually working on the identification of MMP14 in the blood of MM patients. Our preliminary data show that this protein is, indeed, detectable in the peripheral blood of several MM patients, while it is not revealed in healthy controls (data not shown). Molecular analyses of MM are becoming crucial for the tumour comprehension next step that is the identification of molecules that can be used not only for prognostic purposes but to diagnose MM in a very early stage before surgery. The identification of such tumour biomarkers might in dept greatly facilitate surveillance procedures for all the cohorts of patients exposed to asbestos that will be expected increasing in the next 10–15 years.*

## Supporting Information

Table S1 Differentially expressed genes in mesothelioma. The 386 genes retrieved by IPA analysis; for each gene is reported the name and the corresponding Affymetrix_ID, fold change, cellular localization and molecule, and the Entrez Gene ID. Genes identified as putative biomarkers are marked by asterisks.
(0.78 MB DOC)

Table S2 Genes associated to top network functions. All the molecules included in the network are listed in red (over-expressed) or in green (und-erexpressed). Score is the number of eligible molecules in that network. Focus Molecules is the maximun number of network eligible molecules (that is 35).
(0.03 MB DOC)
