# Supplementary material for: Global Gene Expression Profiling Of Human Pleural Mesotheliomas: Identification of Matrix Metalloproteinase 14 (MMP-14) as Potential Tumour Target
Source: PLoS One. 2009 Sep 15;4(9):e7016. doi: 10.1371/journal.pone.0007016 (PMC2737627; doi:10.1371/journal.pone.0007016)
Supplement: Table S1 — Differentially expressed genes in mesothelioma. The 386 genes retrieved by IPA analysis; for each gene is reported the name and the corresponding Affymetrix_ID, fold change, cellular localization and molecule, and the Entrez Gene ID. Genes identified as putative biomarkers are marked by asterisks. (0.78 MB DOC) [file pone.0007016.s001.doc]

| **Name** | **Affymetrix** | **Fold Change** | **Location** | **Type** | **Entrez Gene ID** |
| --- | --- | --- | --- | --- | --- |
| ABCA6 | 217504_at | -2.922 | Plasma Membrane | transporter | 23460 |
| ACSL1 ***** | 207275_s_at | -2.245 | Cytoplasm | enzyme | 2180 |
| ACSS2 | 234312_s_at | -1.455 | Cytoplasm | enzyme | 55902 |
| ACTA2 | 200974_at | -0.527 | Cytoplasm | other | 59 |
| ACTC | 205132_at | -2.134 | Cytoplasm | other | 70 |
| ACTG2 | 202274_at | -0.799 | Cytoplasm | other | 72 |
| ACTR2 | 200727_s_at | 3.577 | Plasma Membrane | other | 10097 |
| ACYP2 | 206833_s_at | -2.892 | Unknown | enzyme | 98 |
| ADD3 | 201753_s_at | -1.797 | Cytoplasm | other | 120 |
| AGRIN | 212285_s_at | 1.697 | Plasma Membrane | other | 375790 |
| AGTR1***** | 205357_s_at | -5.692 | Plasma Membrane | G-protein coupled receptor | 185 |
| AIF1 | 209901_x_at | -1.047 | Nucleus |  | 199 |
| AKAP12 | 231067_s_at | -1.847 | Cytoplasm | transporter | 9590 |
| AKR1C1 | 204151_x_at | -4.391 | Cytoplasm | enzyme | 1645 |
| AKR1C2 | 209699_x_at | -5.264 | Cytoplasm | enzyme | 1646 |
| AKT1 | 207163_s_at | -0.113 | Cytoplasm | kinase | 207 |
| AKT2 | 225471_s_at | 1.366 | Cytoplasm | kinase | 208 |
| ALDH1A1 | 212224_at | -3.702 | Cytoplasm | enzyme | 216 |
| ANGPTL1 | 224339_s_at | -3.17 | Extracellular Space | other | 9068 |
| ANK2 ***** | 202920_at | -3.926 | Plasma Membrane | other | 287 |
| ANKRD32 | 223542_at | 1.737 | Nucleus | transcription regulator | 84250 |
| ANP32A | 201043_s_at | 2.785 | Nucleus | other | 8125 |
| ANXA6 | 200982_s_at | 0.884 | Plasma Membrane | other | 309 |
| AOC3 ***** | 204894_s_at | -3.018 | Plasma Membrane | enzyme | 8639 |
| AOX1 | 205083_at | -5.154 | Cytoplasm | enzyme | 316 |
| AP2B1 | 200612_s_at | 3.588 | Cytoplasm | transporter | 163 |
| APBB2 ***** | 213419_at | 1.51 | Cytoplasm | other | 323 |
| APP ***** | 214953_s_at | 3.137 | Plasma Membrane | other | 351 |
| AQP9 | 205568_at | -5.766 | Plasma Membrane | transporter | 366 |
| ARHGAP6 | 206167_s_at | -3.01 | Cytoplasm | other | 395 |
| ARHGAP26 | 1557688_at | -3.068 | Cytoplasm | other | 23092 |
| ARHGEF3 ***** | 218501_at | -1.876 | Cytoplasm | other | 50650 |
| ARHGEF18 | 213039_at | 0.719 | Cytoplasm | other | 23370 |
| ARID5B | 1561181_at | -3.804 | Nucleus | transcription regulator | 84159 |
| ARRB1 | 222912_at | -1.878 | Cytoplasm | other | 408 |
| ASPM | 219918_s_at | 6 | Unknown | other | 259266 |
| ASS | 207076_s_at | -0.077 | Cytoplasm | enzyme | 445 |
| ATP1A1 | 220948_s_at | 0.509 | Plasma Membrane | transporter | 476 |
| ATP5B | 201322_at | -0.317 | Cytoplasm | transporter | 506 |
| ATP5J ***** | 229127_at | -4.084 | Cytoplasm | transporter | 522 |
| ATRX * | 236778_at | 3.14 | Nucleus | transcription regulator | 546 |
| AURKA ***** | 208079_s_at | 3.564 | Nucleus | kinase | 6790 |
| AZIN1 | 240231_at | -1.775 | Cytoplasm | enzyme | 51582 |
| BAX ***** | 211833_s_at | 2.519 | Cytoplasm | other | 581 |
| BCL6 | 228758_at | -2.272 | Nucleus | transcription regulator | 604 |
| BCL6B | 228311_at | -0.89 | Nucleus | transcription regulator | 255877 |
| BGN ***** | 201262_s_at | 3.27 | Extracellular Space | other | 633 |
| BIRC5 ***** | 202095_s_at | 3.595 | Cytoplasm | other | 332 |
| BTG1  ***** | 243509_at | -3.171 | Nucleus | transcription regulator | 694 |
| BTG2 | 201236_s_at | -2.484 | Nucleus | transcription regulator | 7832 |
| BUB1 (includes EG:699) ***** | 209642_at | 3.884 | Nucleus | kinase | 699 |
| BUB1B | 203755_at | 3.529 | Nucleus | kinase | 701 |
| C5ORF13 | 222344_at | 2.759 | Cytoplasm | other | 9315 |
| C8ORF4 | 218541_s_at | -2.367 | Unknown | other | 56892 |
| C9ORF26 | 209821_at | -3.726 | Nucleus | other | 90865 |
| CAD | 202715_at | 1.706 | Cytoplasm | enzyme | 790 |
| CALD1 | 201615_x_at | 1.333 | Cytoplasm | other | 800 |
| CARHSP1 | 218384_at | 1.376 | Cytoplasm | other | 23589 |
| CASP8 | 213373_s_at | 0.745 | Nucleus | peptidase | 841 |
| CBFB | 206788_s_at | 1.221 | Nucleus | transcription regulator | 865 |
| CBS ***** | 212816_s_at | 3.889 | Cytoplasm | enzyme | 875 |
| CCDC99 ***** | 221685_s_at | 2.009 | Unknown | other | 54908 |
| CCL14 * | 205392_s_at | -4.306 | Extracellular Space | cytokine | 6358 |
| CCL19 | 210072_at | -2.064 | Extracellular Space | cytokine | 6363 |
| CCNA2 ***** | 213226_at | 1.967 | Nucleus | other | 890 |
| CCNB1 ***** | 228729_at | 3.448 | Nucleus | kinase | 891 |
| CCNB2 ***** | 202705_at | 4.083 | Nucleus | other | 9133 |
| CCNE2(includes EG:9134) ***** | 205034_at | 2.326 | Nucleus | other | 9134 |
| CCNG1 | 208796_s_at | -0.502 | Nucleus | other | 900 |
| CCNL2 ***** | 221427_s_at | 1.482 | Nucleus | other | 81669 |
| CCR1 | 205099_s_at | -1.729 | Plasma Membrane | G-protein coupled receptor | 1230 |
| CD34 | 209543_s_at | -3.042 | Plasma Membrane | other | 947 |
| CD36 | 206488_s_at | -3.181 | Plasma Membrane | other | 948 |
| CDC2 ***** | 210559_s_at | 3.28 | Nucleus | kinase | 983 |
| CDCA1 | 223381_at | 4.158 | Nucleus | other | 83540 |
| CDCA3 ***** | 223307_at | 2.93 | Unknown | other | 83461 |
| CDH5 | 204677_at | -1.88 | Plasma Membrane | other | 1003 |
| CDKN1A ***** | 202284_s_at | -0.616 | Nucleus | other | 1026 |
| CDKN2A | 209644_x_at | 0.39 | Nucleus | transcription regulator | 1029 |
| CEBPA | 204039_at | -1.742 | Nucleus | transcription regulator | 1050 |
| CENPA | 204962_s_at | 3.817 | Nucleus | other | 1058 |
| CENPF ***** | 207828_s_at | 4.205 | Nucleus | other | 1063 |
| CENPJ | 223513_at | 1.689 | Nucleus | transcription regulator | 55835 |
| CENPK | 222848_at | 3.078 | Unknown | other | 64105 |
| CFD | 205382_s_at | -3.247 | Extracellular Space | peptidase | 1675 |
| CHEK1 ***** | 238075_at | 2.674 | Nucleus | kinase | 1111 |
| CHES1 | 218031_s_at | -1.643 | Nucleus | transcription regulator | 1112 |
| CHMP4B | 225119_at | 3.058 | Cytoplasm | other | 128866 |
| CIT | 212801_at | 2.506 | Cytoplasm | kinase | 11113 |
| CKAP2 (includes EG:26586) ***** | 218252_at | 1.561 | Cytoplasm | other | 26586 |
| CLEC3B | 205200_at | -3.826 | Extracellular Space | other | 7123 |
| CNAP1 | 201774_s_at | 1.329 | Nucleus | other | 9918 |
| COL10A1 | 217428_s_at | 4.415 | Extracellular Space | other | 1300 |
| COL6A1 ***** | 212940_at | 3.338 | Extracellular Space | other | 1291 |
| COL6A3 | 201438_at | 0.54 | Extracellular Space | other | 1293 |
| COX7A1 | 204570_at | -3.662 | Cytoplasm | enzyme | 1346 |
| CROP | 208835_s_at | 1.475 | Nucleus | other | 51747 |
| CRYAB ***** | 209283_at | -4.099 | Nucleus | other | 1410 |
| CSPG2 | 211571_s_at | 1.884 | Extracellular Space | other | 1462 |
| CTNNB1 | 1570507_at | 1.023 | Nucleus | transcription regulator | 1499 |
| CUGBP2 | 1557422_at | -4.364 | Unknown | other | 10659 |
| CXADR | 1555716_a_at | 1.653 | Plasma Membrane | G-protein coupled receptor | 1525 |
| CXCL1 | 204470_at | -1.831 | Extracellular Space | cytokine | 2919 |
| CXCL2 | 209774_x_at | -3.287 | Extracellular Space | cytokine | 2920 |
| CXCL6 | 206336_at | -4.248 | Extracellular Space | cytokine | 6372 |
| CYBB | 203922_s_at | -1.559 | Cytoplasm | enzyme | 1536 |
| CYFIP2 | 215785_s_at | -0.489 | Cytoplasm | other | 26999 |
| CYP39A1 | 220432_s_at | -3.918 | Cytoplasm | enzyme | 51302 |
| DAPK3 | 203890_s_at | 3.775 | Cytoplasm | kinase | 1613 |
| DARC ***** | 208335_s_at | -4.139 | Plasma Membrane | G-protein coupled receptor | 2532 |
| DHFR ***** | 48808_at | 1.83 | Unknown | enzyme | 1719 |
| DKFZP762E1312 | 218726_at | 3.625 | Unknown | other | 55355 |
| DLG3 | 212727_at | 1.187 | Plasma Membrane | other | 1741 |
| DLG7 ***** | 203764_at | 3.896 | Nucleus | phosphatase | 9787 |
| DMD ***** | 203881_s_at | -2.55 | Plasma Membrane | other | 1756 |
| DNAJC7 | 202416_at | 1.99 | Cytoplasm | other | 7266 |
| DNMT3A | 222640_at | 0.751 | Nucleus | enzyme | 1788 |
| DONSON | 221677_s_at | 1.806 | Unknown | other | 29980 |
| DPT | 213071_at | -5.287 | Extracellular Space | other | 1805 |
| DTL ***** | 218585_s_at | 3.29 | Nucleus | other | 51514 |
| EBF | 244876_at | -3.543 | Nucleus | transcription regulator | 1879 |
| ECT2 | 219787_s_at | 2.396 | Nucleus | other | 1894 |
| EDG1 ***** | 204642_at | -2.029 | Plasma Membrane | G-protein coupled receptor | 1901 |
| EDN1 | 222802_at | -1.275 | Extracellular Space | other | 1906 |
| EFNB1 | 202711_at | 0.087 | Plasma Membrane | other | 1947 |
| EGR1 | 201693_s_at | -3.427 | Nucleus | transcription regulator | 1958 |
| EIF4A1 | 214805_at | -1.291 | Cytoplasm | translation regulator | 1973 |
| ELN | 212670_at | -2.166 | Extracellular Space | other | 2006 |
| EMCN | 219436_s_at | -3.033 | Unknown | other | 51705 |
| EME1 | 234464_s_at | 1.426 | Nucleus | other | 146956 |
| EML2 | 204398_s_at | 1.741 | Cytoplasm | other | 24139 |
| ENO2 | 201313_at | 2.62 | Cytoplasm | enzyme | 2026 |
| EPAS1 ***** | 242868_at | -2.508 | Nucleus | transcription regulator | 2034 |
| EPHB2 ***** | 211165_x_at | 4.061 | Plasma Membrane | kinase | 2048 |
| ERF | 203643_at | 3.273 | Nucleus | transcription regulator | 2077 |
| EZH2 ***** | 203358_s_at | 3.294 | Nucleus | transcription regulator | 2146 |
| F13A1 | 203305_at | -0.443 | Extracellular Space | enzyme | 2162 |
| FABP4 | 235978_at | -4.327 | Cytoplasm | transporter | 2167 |
| FABP5 | 202345_s_at | -1.485 | Cytoplasm | transporter | 2171 |
| FAM105B | 229268_at | 1.393 | Cytoplasm | other | 90268 |
| FBXW11 | 209456_s_at | 1.66 | Cytoplasm | enzyme | 23291 |
| FCGR2B | 210889_s_at | -1.967 | Plasma Membrane | transmembrane receptor | 2213 |
| FGFR3 ***** | 204379_s_at | 3.892 | Plasma Membrane | kinase | 2261 |
| FHL1 | 201539_s_at | -5.309 | Cytoplasm | other | 2273 |
| FILIP1 | 1556325_at | -3.931 | Unknown | other | 27145 |
| FKBP10 | 219249_s_at | 4.076 | Cytoplasm | enzyme | 60681 |
| FMO2 | 228268_at | -3.578 | Cytoplasm | enzyme | 2327 |
| FOXO3A (includes EG:2309) ***** | 242320_at | -1.484 | Nucleus | transcription regulator | 2309 |
| FOXP4 | 227120_at | 2.092 | Nucleus | transcription regulator | 116113 |
| FSTL3 ***** | 203592_s_at | 3.033 | Extracellular Space | other | 10272 |
| FUBP1 | 214093_s_at | 1.041 | Nucleus | transcription regulator | 8880 |
| FUS ***** | 217370_x_at | 3.024 | Nucleus | transcription regulator | 2521 |
| FXR1 | 229519_at | 1.357 | Cytoplasm | other | 8087 |
| FZD7 | 203705_s_at | -2.328 | Plasma Membrane | G-protein coupled receptor | 8324 |
| G0S2 | 213524_s_at | -3.536 | Unknown | other | 50486 |
| G6PD | 202275_at | 1.76 | Cytoplasm | enzyme | 2539 |
| GALNT7 | 222587_s_at | 2.703 | Cytoplasm | enzyme | 51809 |
| GALNT10 | 207357_s_at | 3.653 | Cytoplasm | enzyme | 55568 |
| GALNTL2 | 228501_at | -4.564 | Cytoplasm | enzyme | 117248 |
| GANAB | 211934_x_at | 1.205 | Cytoplasm | enzyme | 23193 |
| GATAD2A | 234294_x_at | 2.724 | Nucleus | transcription regulator | 54815 |
| GINS1 | 206102_at | 3.7 | Unknown | other | 9837 |
| GNB1 | 200745_s_at | 1.025 | Plasma Membrane | enzyme | 2782 |
| GNG11 | 204115_at | -2.103 | Plasma Membrane | enzyme | 2791 |
| GPI | 208308_s_at | -0.072 | Extracellular Space | enzyme | 2821 |
| GPS2 | 209350_s_at | -0.439 | Nucleus | other | 2874 |
| GSK3B | 209945_s_at | 1.19 | Nucleus | kinase | 2932 |
| GUCY1A3 | 229530_at | -2.152 | Cytoplasm | enzyme | 2982 |
| HCAP-D3 | 212789_at | 1.585 | Nucleus | other | 23310 |
| HCAP-G | 218663_at | 3.412 | Nucleus | other | 64151 |
| HDAC1 (includes EG:3065) | 201209_at | -0.075 | Nucleus | transcription regulator | 3065 |
| HELLS ***** | 227350_at | 2.756 | Nucleus | enzyme | 3070 |
| HGF | 209960_at | -1.099 | Extracellular Space | growth factor | 3082 |
| HLA-DQA1 | 213831_at | -4.063 | Plasma Membrane | transmembrane receptor | 3117 |
| HMGB3 | 225601_at | 4.541 | Nucleus | other | 3149 |
| HMGCS1 | 205822_s_at | 2.739 | Cytoplasm | enzyme | 3157 |
| HMGN2 | 208668_x_at | 0.199 | Nucleus | other | 3151 |
| HMMR ***** | 207165_at | 4.409 | Plasma Membrane | other | 3161 |
| HNRPH1 | 213470_s_at | 1.836 | Nucleus | other | 3187 |
| HOXA5 ***** | 213844_at | -3.611 | Nucleus | transcription regulator | 3202 |
| HRAS | 212983_at | 0.615 | Plasma Membrane | enzyme | 3265 |
| HSPA4 | 211016_x_at | 1.998 | Cytoplasm | other | 3308 |
| HSPB2 | 205824_at | -2.947 | Cytoplasm | other | 3316 |
| HSPB6 | 214767_s_at | -5.037 | Cytoplasm | other | 126393 |
| HSPG2 (includes EG:3339) | 201655_s_at | 0.063 | Plasma Membrane | other | 3339 |
| ICAM2 ***** | 204683_at | -2.464 | Plasma Membrane | other | 3384 |
| ID2 ***** | 213931_at | -2.275 | Nucleus | transcription regulator | 3398 |
| IGF1 | 209540_at | -1.726 | Extracellular Space | growth factor | 3479 |
| IL16 | 209827_s_at | -1.209 | Extracellular Space | cytokine | 3603 |
| IL1B | 205067_at | -2.109 | Extracellular Space | cytokine | 3553 |
| IL8RB ***** | 207008_at | -3.287 | Plasma Membrane | G-protein coupled receptor | 3579 |
| INPPL1 ***** | 201598_s_at | 1.124 | Cytoplasm | phosphatase | 3636 |
| IRX3 (includes EG:79191) | 229638_at | 2.908 | Nucleus | transcription regulator | 79191 |
| ITGA7 ***** | 216331_at | -2.819 | Plasma Membrane | other | 3679 |
| ITGB4 | 204989_s_at | 2.098 | Plasma Membrane | transmembrane receptor | 3691 |
| ITPKB | 203723_at | -1.444 | Cytoplasm | kinase | 3707 |
| JUB | 1553764_a_at | 2.091 | Plasma Membrane | other | 84962 |
| KIAA0101 | 202503_s_at | 3.695 | Nucleus | other | 9768 |
| KIAA1794 ***** | 213007_at | 3.472 | Unknown | other | 55215 |
| KIF11 | 204444_at | 3.835 | Nucleus | other | 3832 |
| KIF14 ***** | 206364_at | 4.127 | Cytoplasm | other | 9928 |
| KIF23 ***** | 204709_s_at | 4.061 | Cytoplasm | other | 9493 |
| KIF20A ***** | 218755_at | 3.91 | Cytoplasm | transporter | 10112 |
| KIF4A ***** | 218355_at | 3.737 | Nucleus | other | 24137 |
| KIFC1 | 209680_s_at | 2.735 | Nucleus | other | 3833 |
| KITLG (includes EG:4254) | 226534_at | -0.095 | Extracellular Space | growth factor | 4254 |
| KLF10 | 244447_at | -1.714 | Nucleus | transcription regulator | 7071 |
| KNTC1 | 206316_s_at | 2.366 | Nucleus | other | 9735 |
| KNTC2 | 204162_at | 2.45 | Nucleus | other | 10403 |
| KRT5 | 201820_at | 1.557 | Cytoplasm | other | 3852 |
| LAMA1 | 227048_at | 2.688 | Extracellular Space | other | 284217 |
| LAMA4 ***** | 216081_at | -2.425 | Extracellular Space | enzyme | 3910 |
| LAMA5 ***** | 210150_s_at | 2.075 | Extracellular Space | other | 3911 |
| LAMB3 | 209270_at | 3.476 | Extracellular Space | transporter | 3914 |
| LAMC2 ***** | 202267_at | 5.579 | Extracellular Space | other | 3918 |
| LDB2 | 232852_at | -2.622 | Nucleus | transcription regulator | 9079 |
| LEF1 ***** | 221558_s_at | 2.213 | Nucleus | transcription regulator | 51176 |
| LMNB1 ***** | 203276_at | 2.114 | Nucleus | other | 4001 |
| LMO2 | 204249_s_at | -1.38 | Nucleus | other | 4005 |
| LPL | 203548_s_at | -4.813 | Extracellular Space | enzyme | 4023 |
| LTBR | 203005_at | 0.636 | Plasma Membrane | transmembrane receptor | 4055 |
| LTC4S | 206480_at | 0.154 | Cytoplasm | enzyme | 4056 |
| LUZP5 | 219588_s_at | 1.975 | Nucleus | other | 54892 |
| MAD2L1 ***** | 1554768_a_at | 3.554 | Nucleus | other | 4085 |
| MAP4K5 | 203553_s_at | 1.253 | Cytoplasm | kinase | 11183 |
| MBD2 | 202484_s_at | -0.381 | Nucleus | transcription regulator | 8932 |
| MCM2 ***** | 202107_s_at | 2.159 | Nucleus | enzyme | 4171 |
| MCM4 | 222036_s_at | 1.829 | Nucleus | enzyme | 4173 |
| MDK ***** | 209035_at | 3.595 | Extracellular Space | growth factor | 4192 |
| MELK | 204825_at | 3.922 | Cytoplasm | kinase | 9833 |
| MEOX1 | 205619_s_at | -3.64 | Nucleus | transcription regulator | 4222 |
| MITF ***** | 207233_s_at | -2.232 | Nucleus | transcription regulator | 4286 |
| MKI67 ***** | 212022_s_at | 4.068 | Nucleus | other | 4288 |
| MMP11 ***** | 203878_s_at | 2.51 | Extracellular Space | peptidase | 4320 |
| MMP14 ***** | 202827_s_at | 6.19 | Extracellular Space | peptidase | 4323 |
| MTA3 | 223311_s_at | 0.871 | Nucleus | other | 57504 |
| MUC1 | 213693_s_at | 1.849 | Plasma Membrane | other | 4582 |
| MUS81 | 218463_s_at | 0.748 | Nucleus | enzyme | 80198 |
| MYC | 202431_s_at | -0.327 | Nucleus | transcription regulator | 4609 |
| MYCT1 | 231947_at | -2.141 | Nucleus | other | 80177 |
| NCOA6IP | 238346_s_at | 1.853 | Nucleus | transcription regulator | 96764 |
| NEK2 | 204641_at | 3.761 | Nucleus | kinase | 4751 |
| NF1 | 212676_at | 1.077 | Cytoplasm | other | 4763 |
| NFKBIZ | 223218_s_at | -1.155 | Nucleus | transcription regulator | 64332 |
| NGEF | 227240_at | 1.865 | Cytoplasm | other | 25791 |
| NIPBL ***** | 242352_at | 3.19 | Nucleus | other | 25836 |
| NOSIP | 217950_at | -0.363 | Cytoplasm | other | 51070 |
| NR1H2 | 218215_s_at | -0.254 | Nucleus | ligand-dependent nuclear receptor | 7376 |
| NR3C1 ***** | 211671_s_at | -1.207 | Nucleus | ligand-dependent nuclear receptor | 2908 |
| NR4A2 | 204621_s_at | -2.966 | Nucleus | ligand-dependent nuclear receptor | 4929 |
| NRN1 | 218625_at | -5.438 | Cytoplasm | other | 51299 |
| NRP2 | 225566_at | 2.316 | Plasma Membrane | kinase | 8828 |
| NTRK2 ***** | 221796_at | -4.383 | Plasma Membrane | kinase | 4915 |
| NUSAP1 | 219978_s_at | 3.368 | Nucleus | other | 51203 |
| NUTF2 | 202397_at | 1.274 | Nucleus | transporter | 10204 |
| OGG1 | 205760_s_at | -0.166 | Nucleus | enzyme | 4968 |
| ORC6L | 219105_x_at | 2.054 | Nucleus | other | 23594 |
| PAIP1 | 208051_s_at | 2.573 | Cytoplasm | translation regulator | 10605 |
| PBX1 | 233273_at | 1.787 | Nucleus | transcription regulator | 5087 |
| PCDH7 | 210273_at | 0.267 | Plasma Membrane | other | 5099 |
| PDE1A | 231213_at | -2.948 | Cytoplasm | enzyme | 5136 |
| PDE4B ***** | 203708_at | -2.482 | Cytoplasm | enzyme | 5142 |
| PECAM1 ***** | 208981_at | -2.228 | Plasma Membrane | other | 5175 |
| PFDN6 | 233588_x_at | 1.733 | Cytoplasm | other | 10471 |
| PIP5K1A | 207391_s_at | 1.419 | Cytoplasm | kinase | 8394 |
| PLAGL1 | 207002_s_at | -1.538 | Nucleus | transcription regulator | 5325 |
| PLCL2 ***** | 216218_s_at | -1.952 | Cytoplasm | enzyme | 23228 |
| PLN | 204939_s_at | -5.017 | Cytoplasm | other | 5350 |
| PLXNA1 | 221538_s_at | 1.284 | Plasma Membrane | transmembrane receptor | 5361 |
| PNN | 212036_s_at | 1.178 | Plasma Membrane | other | 5411 |
| PNRC1 | 209034_at | -1.232 | Nucleus | other | 10957 |
| PODXL | 201578_at | 0.342 | Plasma Membrane | other | 5420 |
| POLR2J2 | 231406_at | 2.526 | Nucleus | transcription regulator | 246721 |
| PPARA ***** | 223437_at | -1.951 | Nucleus | ligand-dependent nuclear receptor | 5465 |
| PPP1R12B | 201957_at | -2.52 | Cytoplasm | phosphatase | 4660 |
| PPP1R14B | 212680_x_at | 1.149 | Unknown | phosphatase | 26472 |
| PPP1R3C | 204284_at | -3.193 | Unknown | phosphatase | 5507 |
| PRC1 | 218009_s_at | 3.551 | Nucleus | other | 9055 |
| PRELP | 204223_at | -2.838 | Extracellular Space | other | 5549 |
| PRKCA | 213093_at | 0.234 | Cytoplasm | kinase | 5578 |
| PRPS1 | 208447_s_at | 1.906 | Unknown | kinase | 5631 |
| PSMC3IP | 213951_s_at | 1.232 | Nucleus | other | 29893 |
| PTGDS ***** | 212187_x_at | -3.473 | Cytoplasm | enzyme | 5730 |
| PTGER2 | 206631_at | -1.112 | Plasma Membrane | G-protein coupled receptor | 5732 |
| PTK7 | 207011_s_at | 2.197 | Plasma Membrane | kinase | 5754 |
| PTPN2 | 1557193_at | 1.392 | Cytoplasm | phosphatase | 5771 |
| PTPRB | 230250_at | -1.799 | Plasma Membrane | phosphatase | 5787 |
| RACGAP1 ***** | 222077_s_at | 2.126 | Cytoplasm | transporter | 29127 |
| RAD51AP1 | 204146_at | 2.336 | Nucleus | other | 10635 |
| RAD54B | 219494_at | 2.371 | Nucleus | enzyme | 25788 |
| RAI14 | 202052_s_at | 2.01 | Nucleus | transcription regulator | 26064 |
| RB1 | 203132_at | 0.279 | Nucleus | transcription regulator | 5925 |
| RBBP6 | 223802_s_at | 1.463 | Nucleus | other | 5930 |
| RBL2 | 212331_at | 0.271 | Nucleus | other | 5934 |
| RBP4 | 219140_s_at | -4.593 | Extracellular Space | transporter | 5950 |
| RCAN2 | 203498_at | -2.553 | Unknown | other | 10231 |
| RELA | 201783_s_at | 0.314 | Nucleus | transcription regulator | 5970 |
| RGS5 | 209070_s_at | -2.752 | Plasma Membrane | other | 8490 |
| RHOD | 209885_at | 1.193 | Cytoplasm | enzyme | 29984 |
| RPS6KA5 (includes EG:9252) ***** | 204633_s_at | -2.341 | Nucleus | kinase | 9252 |
| RPSA | 213801_x_at | 0.019 | Plasma Membrane | transmembrane receptor | 3921 |
| RRM2 | 209773_s_at | 3.864 | Nucleus | enzyme | 6241 |
| RSF1 | 222540_s_at | 1.656 | Nucleus | transcription regulator | 51773 |
| RTKN | 225150_s_at | 0.067 | Cytoplasm | other | 6242 |
| RUNX1T1 | 205529_s_at | -1.589 | Nucleus | transcription regulator | 862 |
| RXRB | 209148_at | 0.006 | Nucleus | ligand-dependent nuclear receptor | 6257 |
| SASH1 | 41644_at | -1.943 | Unknown | other | 23328 |
| SCAMP1 | 212417_at | 1.545 | Cytoplasm | transporter | 9522 |
| SCG2 | 204035_at | 2.217 | Extracellular Space | other | 7857 |
| SDC4 | 202071_at | 1.34 | Plasma Membrane | other | 6385 |
| SELL ***** | 204563_at | -2.69 | Plasma Membrane | other | 6402 |
| SEMA3G | 219689_at | -3.889 | Unknown | other | 56920 |
| SEMA4C | 219039_at | 1.851 | Plasma Membrane | other | 54910 |
| 9-Sep | 208657_s_at | 2.424 | Cytoplasm | enzyme | 10801 |
| SFN | 33323_r_at | 5.399 | Cytoplasm | other | 2810 |
| SH2B2 | 205367_at | -0.813 | Cytoplasm | other | 10603 |
| SHB | 204657_s_at | 2.512 | Unknown | other | 6461 |
| SHC1 | 201469_s_at | 1.591 | Cytoplasm | other | 6464 |
| SHCBP1 | 219493_at | 3.559 | Unknown | other | 79801 |
| SIAHBP1 | 209899_s_at | 0.57 | Nucleus | other | 22827 |
| SLC20A1 | 201920_at | 1.03 | Plasma Membrane | transporter | 6574 |
| SLC25A4 | 202825_at | -1.341 | Cytoplasm | transporter | 291 |
| SLC2A1 | 201250_s_at | 2.737 | Plasma Membrane | transporter | 6513 |
| SLC8A1 | 235518_at | -1.183 | Plasma Membrane | transporter | 6546 |
| SMARCA4 ***** | 213720_s_at | 1.84 | Nucleus | transcription regulator | 6597 |
| SMARCB1 | 212167_s_at | 0.496 | Nucleus | other | 6598 |
| SMC2 | 204240_s_at | 2.001 | Nucleus | transporter | 10592 |
| SMC4 | 201663_s_at | 2.586 | Nucleus | transporter | 10051 |
| SNRP70 | 201221_s_at | 0.55 | Nucleus | other | 6625 |
| SOCS2 | 203373_at | -2.101 | Cytoplasm | other | 8835 |
| SORBS1 | 218087_s_at | -3.673 | Plasma Membrane | other | 10580 |
| SOX4 ***** | 213668_s_at | 2.863 | Nucleus | transcription regulator | 6659 |
| SOX7 | 228698_at | -2.783 | Nucleus | transcription regulator | 83595 |
| SPAG5 | 203145_at | 2.501 | Nucleus | peptidase | 10615 |
| SPARCL1 | 200795_at | -1.461 | Extracellular Space | other | 8404 |
| SPEN | 201996_s_at | 2.201 | Nucleus | transcription regulator | 23013 |
| SPINT2 | 210715_s_at | 1.98 | Extracellular Space | other | 10653 |
| SPRY1 | 212558_at | -2.616 | Plasma Membrane | other | 10252 |
| STIL | 205339_at | 2.338 | Nucleus | other | 6491 |
| STK11 | 231017_at | -0.121 | Cytoplasm | kinase | 6794 |
| STK36 ***** | 234005_x_at | 1.917 | Unknown | kinase | 27148 |
| STMN1 | 200783_s_at | 1.209 | Cytoplasm | other | 3925 |
| SYNPO2 ***** | 225894_at | -6.002 | Cytoplasm | other | 171024 |
| TACC1 ***** | 242290_at | -2.018 | Nucleus | other | 6867 |
| TBL1X | 213400_s_at | -0.052 | Plasma Membrane | other | 6907 |
| TBL1XR1 | 222633_at | 2.06 | Plasma Membrane | enzyme | 79718 |
| TBX2 | 40560_at | -0.576 | Nucleus | transcription regulator | 6909 |
| TCF3 | 213811_x_at | 1.217 | Nucleus | transcription regulator | 6929 |
| TCF19 | 223274_at | 2.163 | Nucleus | transcription regulator | 6941 |
| TEK | 206702_at | -1.802 | Plasma Membrane | kinase | 7010 |
| TFAP2A | 204653_at | 5.488 | Nucleus | transcription regulator | 7020 |
| TGFB1 | 203085_s_at | 0.684 | Extracellular Space | growth factor | 7040 |
| TGFBR3 ***** | 204731_at | -3.748 | Plasma Membrane | kinase | 7049 |
| THOC4 | 226319_s_at | 2.652 | Nucleus | transcription regulator | 10189 |
| TIMELESS | 203046_s_at | 2.139 | Nucleus | other | 8914 |
| TIMP4 | 206243_at | -3.03 | Extracellular Space | other | 7079 |
| TK1 | 1554408_a_at | 3.341 | Cytoplasm | kinase | 7083 |
| TNPO1 | 225766_s_at | 1.582 | Nucleus | transporter | 3842 |
| TOM1 | 202807_s_at | -0.92 | Cytoplasm | transporter | 10043 |
| TOMM34 | 226670_s_at | 3.103 | Cytoplasm | other | 10953 |
| TOP2A ***** | 201291_s_at | 5.569 | Nucleus | enzyme | 7153 |
| TOPBP1 | 202633_at | 1.255 | Nucleus | other | 11073 |
| TPM1 | 206117_at | -2.431 | Cytoplasm | other | 7168 |
| TPR ***** | 1557227_s_at | 3.847 | Nucleus | other | 7175 |
| TPT1 | 216520_s_at | -0.196 | Cytoplasm | other | 7178 |
| TPX2 | 210052_s_at | 4.401 | Nucleus | other | 22974 |
| TRAF4 ***** | 202871_at | 2.662 | Cytoplasm | other | 9618 |
| TRIB3 ***** | 218145_at | 2.984 | Nucleus | kinase | 57761 |
| TSPAN7 ***** | 202242_at | -4.176 | Plasma Membrane | other | 7102 |
| U2AF1 | 202858_at | 1.292 | Nucleus | other | 7307 |
| UBE2C ***** | 202954_at | 3.139 | Cytoplasm | enzyme | 11065 |
| UBE2I | 213535_s_at | 1.328 | Nucleus | enzyme | 7329 |
| UBE2S | 202779_s_at | 2.949 | Unknown | enzyme | 27338 |
| UGDH | 203343_at | 0.074 | Nucleus | enzyme | 7358 |
| UHRF1 | 225655_at | 4.299 | Nucleus | transcription regulator | 29128 |
| VEGF | 210513_s_at | 1.657 | Extracellular Space | growth factor | 7422 |
| VPS4A | 217913_at | -0.228 | Cytoplasm | other | 27183 |
| VWF ***** | 202112_at | -1.9 | Extracellular Space | other | 7450 |
| WASL ***** | 205809_s_at | 3.76 | Cytoplasm | other | 8976 |
| WDR90 | 227894_at | 1.775 | Unknown | other | 197335 |
| WT1 ***** | 206067_s_at | -2.272 | Nucleus | transcription regulator | 7490 |
| WTAP | 1558783_at | -1.832 | Nucleus | other | 9589 |
| YWHAG | 222985_at | -0.501 | Cytoplasm | other | 7532 |
| ZBTB16 | 244697_at | -1.359 | Nucleus | transcription regulator | 7704 |
| ZFYVE16 | 1554638_at | -1.253 | Nucleus | transporter | 9765 |
| ZWINT (includes EG:11130) | 204026_s_at | 2.961 | Nucleus | other | 11130 |
