# Supplementary material for: Global Gene Expression Profiling Of Human Pleural Mesotheliomas: Identification of Matrix Metalloproteinase 14 (MMP-14) as Potential Tumour Target
Source: PLoS One. 2009 Sep 15;4(9):e7016. doi: 10.1371/journal.pone.0007016 (PMC2737627; doi:10.1371/journal.pone.0007016)
Supplement: Table S2 — Genes associated to top network functions. All the molecules included in the network are listed in red (over-expressed) or in green (und-erexpressed). Score is the number of eligible molecules in that network. Focus Molecules is the maximun number of network eligible molecules (that is 35). (0.03 MB DOC) [file pone.0007016.s002.doc]

| **Top functions** | **Molecules in network** | **Score** | **Focus Molecules** |
| --- | --- | --- | --- |
| *Cell Cycle*  *Cancer*  *Cell Death* | **APBB2**  **APP**  **AURKA**  **BAX**  **BGN**  **BIRC5**  **BUB1B**  **CCNA2**  **CCNB1**  **CCNB2**  **CCNL2**  **CDC2**  **CHEK1**  **DHFR**  **DLG7**  **DPT**  **EBF**  **FOXO3A**  **HMMR**  **ID2**  **JUB**  **LAMA4**  **MAD2L1**  **MCM2**  **MCM4**  **NEK2**  **NR3C1**  **NTRK2**  **TCF3**  **TFAP2A**  **TOP2A**  **TPX2**  **UBE2C**  **UBE2I**  **VWF** | 58 | 35 |
| *Cell Cycle*  *Cellular Assembly and Organization*  *DNA Replication Recombination and Repair* | **BRRN1**  **CDCA1**  **CIT**  **CNAP1**  **E2F4**  **ECT2**  **GINS1**  **HCAP-D3**  **HCAP-G**  **HCAP-H2**  **HMGB3**  **HSPG2**  **KIF14**  **KIF23**  **KIF4A**  **KIFC1**  **KNTC2**  **LUZP5**  **NEK2**  **ORC1L**  **ORC6L**  **PRC1**  **PRELP**  **RACGAP1**  **SASH1**  **SMC2**  **SMC4**  **SPBC24**  **SPBC25**  **SPRY1**  **TERT**  **WDR90**  **WT1**  **WTAP**  **ZWINT** | 27 | 22 |
